# Supplementary material for: Elevated microglial oxidative phosphorylation and phagocytosis stimulate post-stroke brain remodeling and cognitive function recovery in mice
Source: Commun Biol. 2022 Jan 11;5:35. doi: 10.1038/s42003-021-02984-4 (PMC8752825; doi:10.1038/s42003-021-02984-4)
Supplement: Supplementary file 2 — Supplemental Materials [file 42003_2021_2984_MOESM2_ESM.pdf]

SUPPLEMENTARY MATERIALS

Supplementary Figure 1. Changes of survival rates and body weights in WT or *Nhe1* cKO mice.

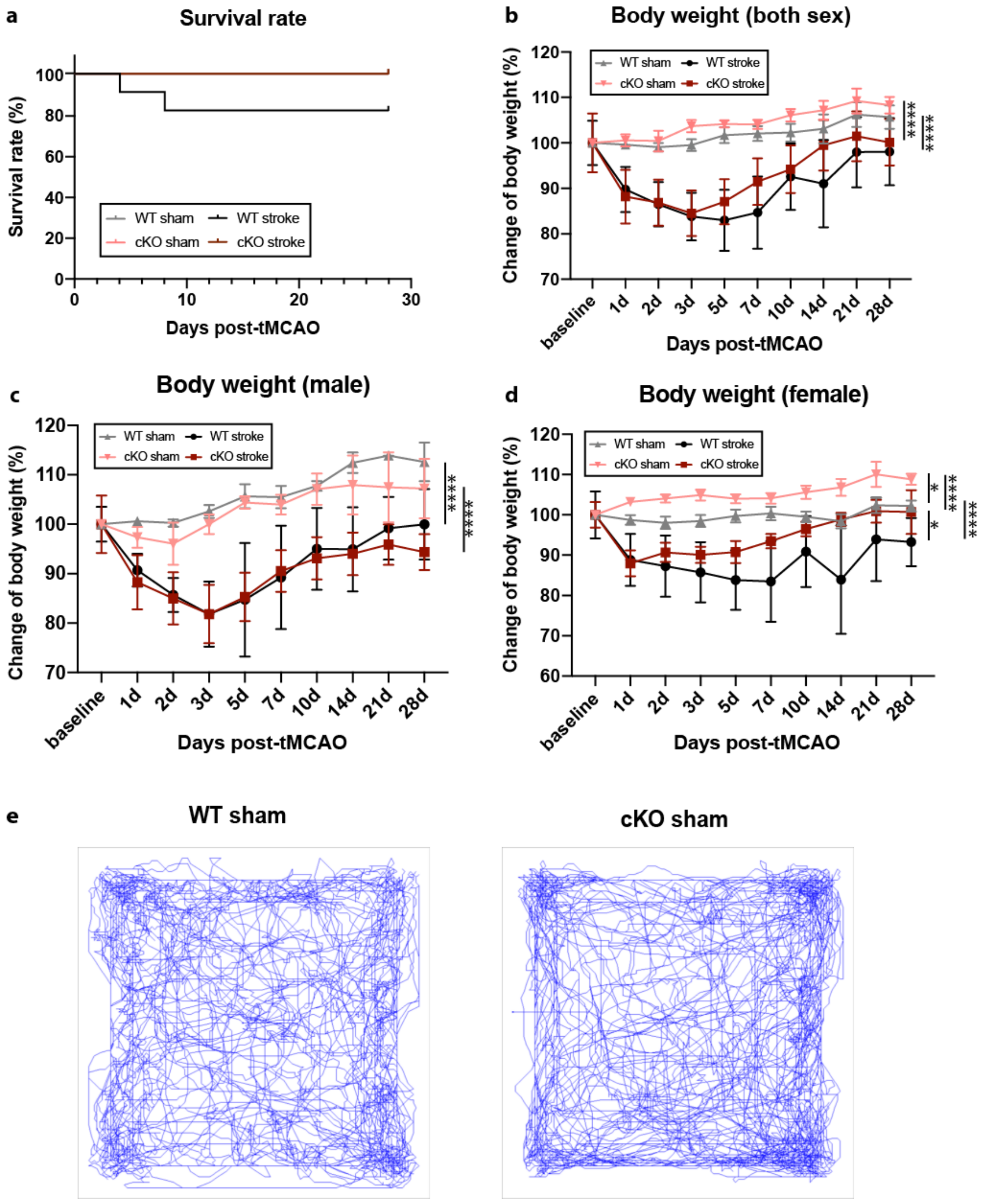

a. Survival curves of WT and *Nhe1* cKO mice during 1-28 d post-sham or stroke procedures. N = 9-12. **b.** Body weight changes of WT and *Nhe1* cKO mice. Same cohort of mice as in A. **c-d.** Body weight changes of male or female WT and *Nhe1* cKO mice. Same cohort of mice as in A. Female cKO mice showed increased recovery of body weight compared to female WT mice, either after sham or stroke. ** $p < 0.05$, **** $p < 0.0001$. **e.** Representative paths of WT and cKO mice underwent sham procedures showed similar traveled distance in the open field test over a 60-min duration.

Supplementary Figure 2. Characterizations of MACS-isolated microglia with transcription markers.

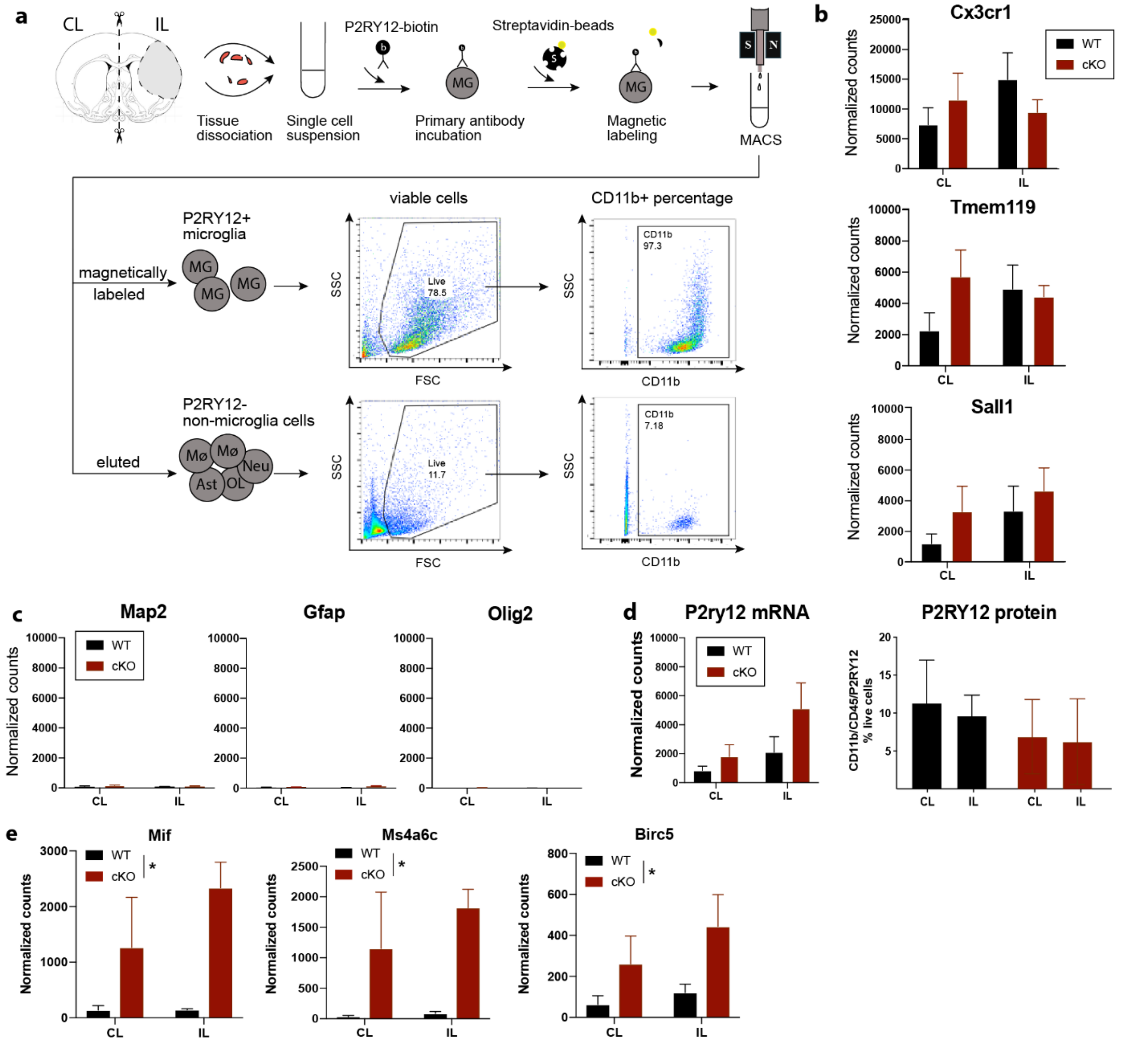

a. Verification of microglial viability and purity in the eluted P2RY12^{+} and P2RY12^{-} populations using flow cytometry. **b.** Transcriptome markers for microglia in the RNAseq analysis. N=3. **c.** Transcriptome markers for other cell types (neurons, astrocytes, oligodendrocytes) in the RNAseq analysis. **d.** Validation of *P2ry12* mRNA expression in RNAseq analysis, and

$CD11b^+CD45^+P2RY12^+$ microglial population using flow cytometry. N=3. **e.** cKO microglia with elevated expression of early developmental microglia transcriptomes. * $p < 0.05$.

Supplementary Figure 3. Transcriptomic analysis of *Nhe1* cKO microglia in the non-lesion CL hemisphere after stroke.

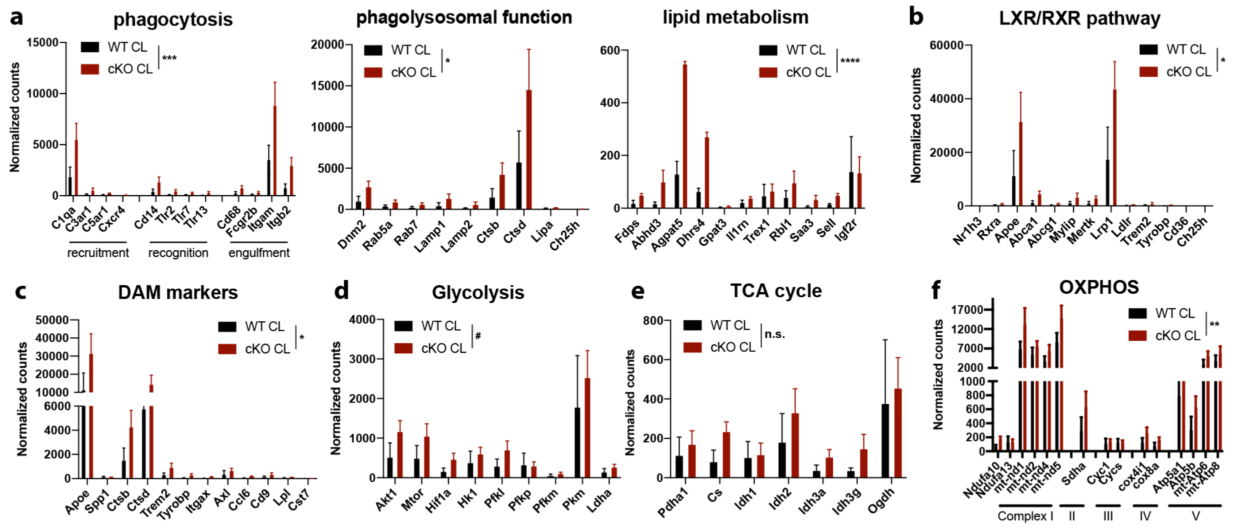

Transcriptome analysis of genes relating to **a.** phagocytosis processes (recruitment, recognition and engulfment), phagolysosomal function, and lipid metabolism; **b.** LXR/RXR pathway activation; **c.** disease-associated microglia markers; **d-f.** energy metabolism processes, including glycolysis, TCA cycle, and oxidative phosphorylation in microglia from the CL hemispheres of WT and cKO brains at 3 d post-stroke. N=3. # $p < 0.1$, * $p < 0.05$, ** $p < 0.01$, *** $p < 0.001$, **** $p < 0.0001$.

Supplementary Figure 4. *Nhe1* cKO brains exhibited increased lysosomal activity of dendritic spines after stroke.

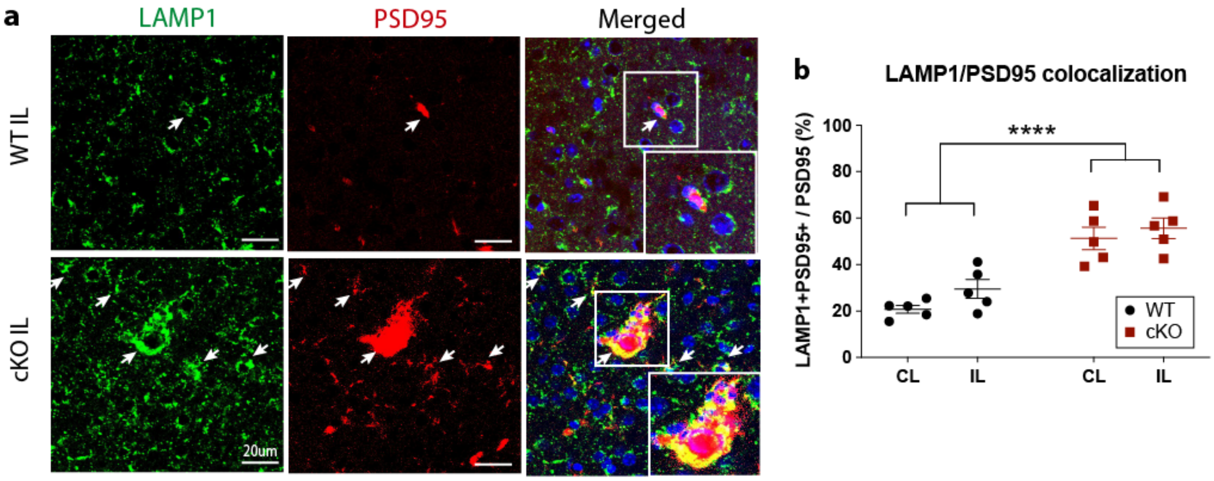

a. Representative staining images of LAMP1, PSD95, and DAPI from the IL peri-lesion area at 3 d post-stroke. Arrows: LAMP1^{+}/PSD95^{+} colocalizing cells. **b.** Quantitative analysis of LAMP1^{+}/PSD95^{+} colocalizing cells. N = 5 animals, 3-8 images per area (CL or IL) per animal.

Supplementary Figure 5. Myeloid and T cells in the spleen and their infiltration in the brain at 3 d post-stroke.

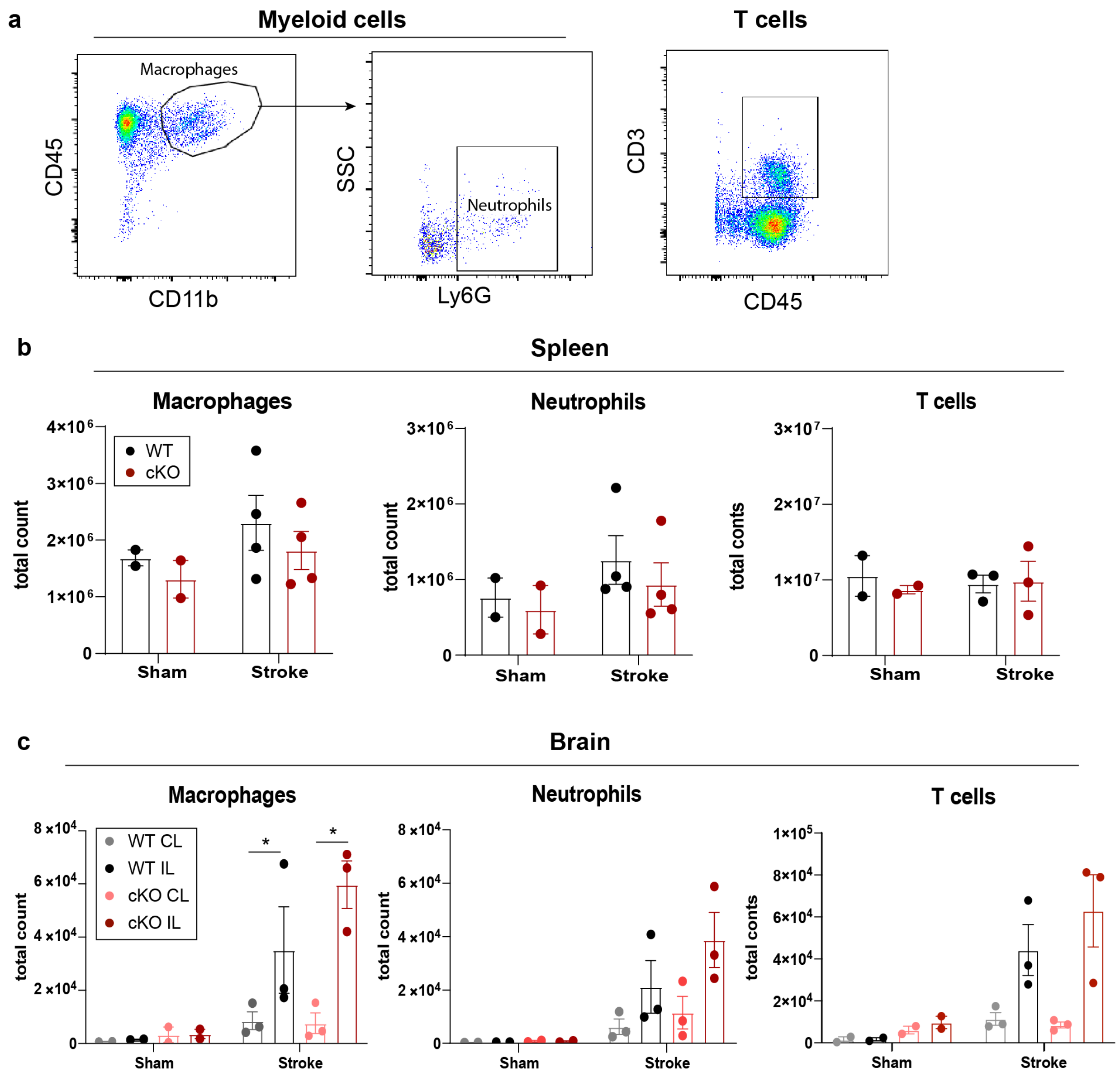

a. Representative gating strategy of CD11b^{+}CD45^{hi}P2RY12^{-} macrophages, CD11b^{+}CD45^{hi}Ly6G^{+} neutrophils, and CD3^{+} T cells from the brain or spleen of WT and cKO mice at 3 d post-sham and post-stroke using flow cytometry. **b.** Total count of macrophages, neutrophils, and T cells within live singlets in the spleens of post-sham or post-stroke animals. **c.** Total count of macrophages,

neutrophils, and T cells within live singlets in the non-lesion CL hemispheres and IL hemispheres post-sham or post-stroke. Data are mean ± SEM, N = 3-4. * $p < 0.05$.

Supplementary Figure 6. Extracellular acidification rate (ECAR) profiles of naïve WT and cKO mice.

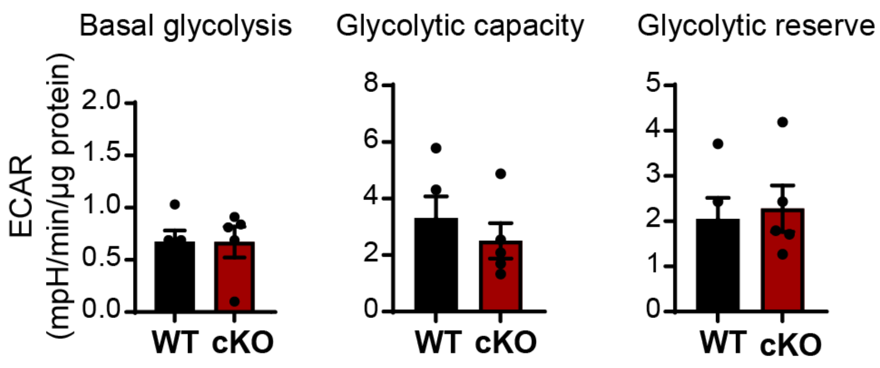

Extracellular acidification rate (ECAR) measured by Seahorse Extracellular Flux Analyzer in isolated P2RY12$^+$ microglial cells from naïve WT (n=2 mice) and cKO mice (n=2 mice). The P2RY12$^+$ cells from 2 WT and 2 cKO mice were pooled to run n=5 repetitive measurements.

Supplementary Figure 7. Changes of CC white matter myelination in WT and *Nhe1* cKO mice after stroke.

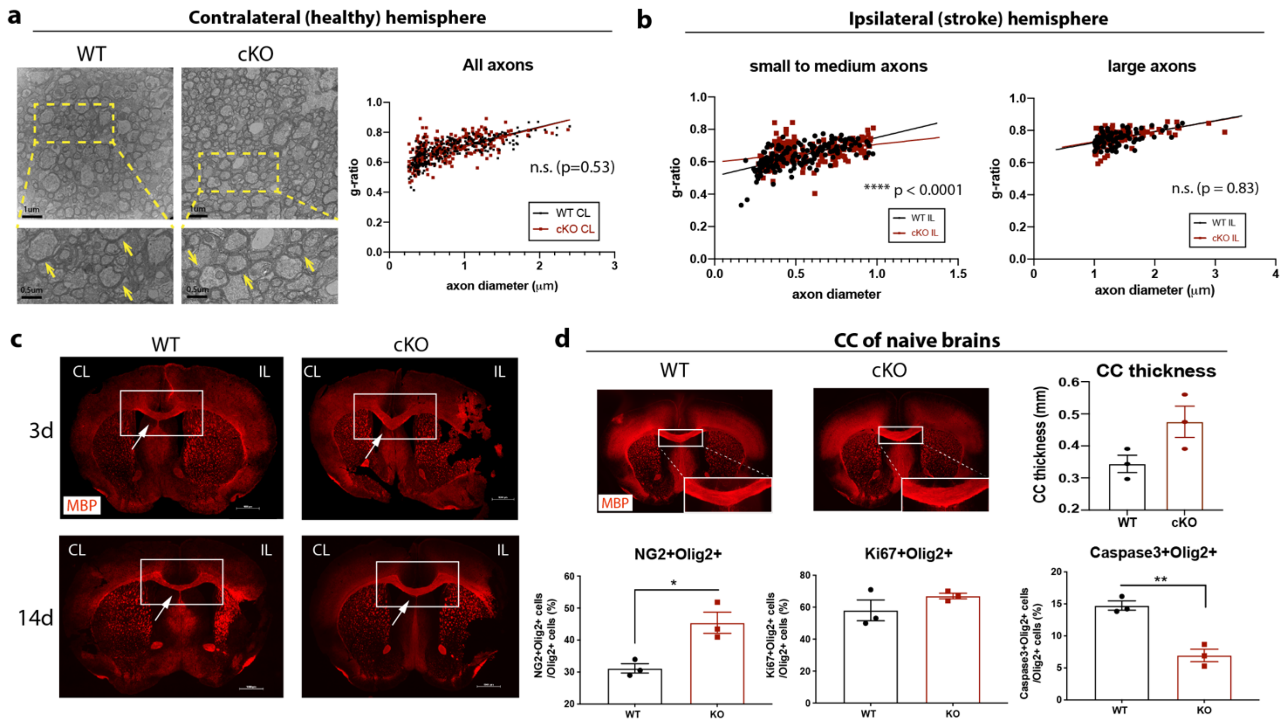

a. Representative images and g-ratio from axons in the medial corpus callosum (CC) by transmission electron microscopy (TEM) in the CL hemispheres of WT and cKO mice. Slopes from the linear regressions in WT or cKO mice were compared. N=4 animals. **b.** G-ratio from small to medium sized or large sized axons in the medial CC in the IL hemispheres of WT and cKO mice. Same cohort of mice as in A. **c.** Whole brain images at the same bregma level showing increased CC thickness in *Nhe1* cKO mice at 3 and 14 d post-stroke by MBP staining. N=3-5. **d.** CC thickness, and oligodendrogenesis in the naïve WT or cKO brains. N=3. * $p < 0.05$, ** $p <$ 0,01, **** $p < 0.0001$.

Supplementary Figure 8. Changes of EC white matter myelination in WT and *Nhe1* cKO mice after stroke.

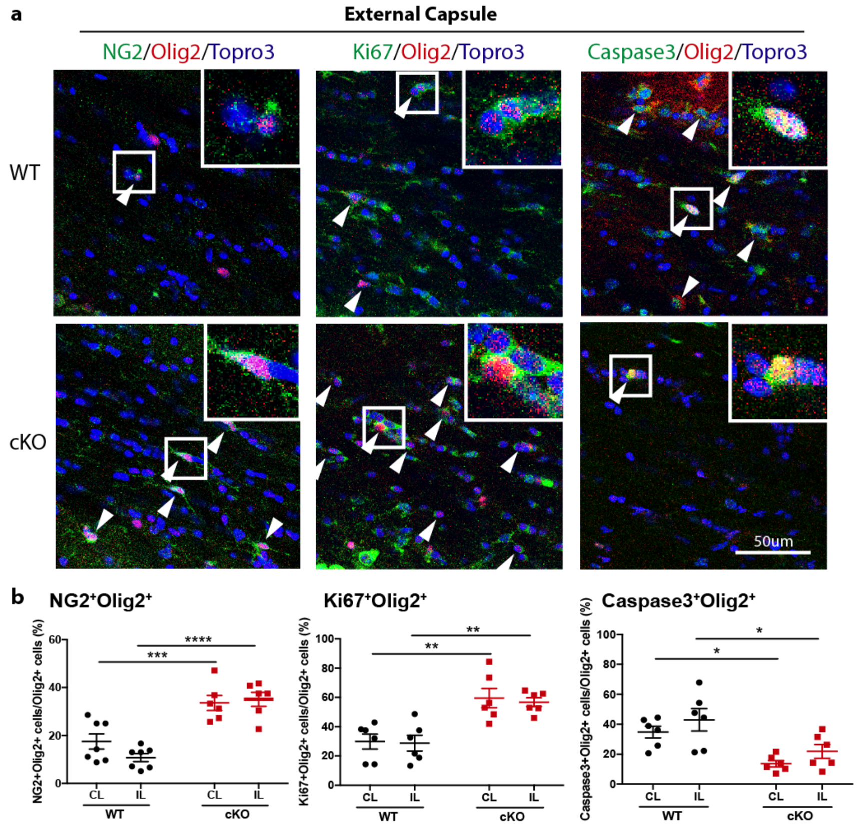

a-b. Representative images and quantitative analysis of NG2, Ki67, and Caspase-3 staining colocalized with Olig2 in external capsules (EC) from CL or IL hemispheres of WT or *Nhe1* cKO mice at 3 d post-stroke. N=6-7. Data are mean ± SEM. * $p < 0.05$, ** $p < 0.01$, *** $p < 0.001$, **** $p < 0.0001$.

Supplementary Figure 9. Stroke-induced transcriptomic changes in WT and *Nhe1* cKO microglia.

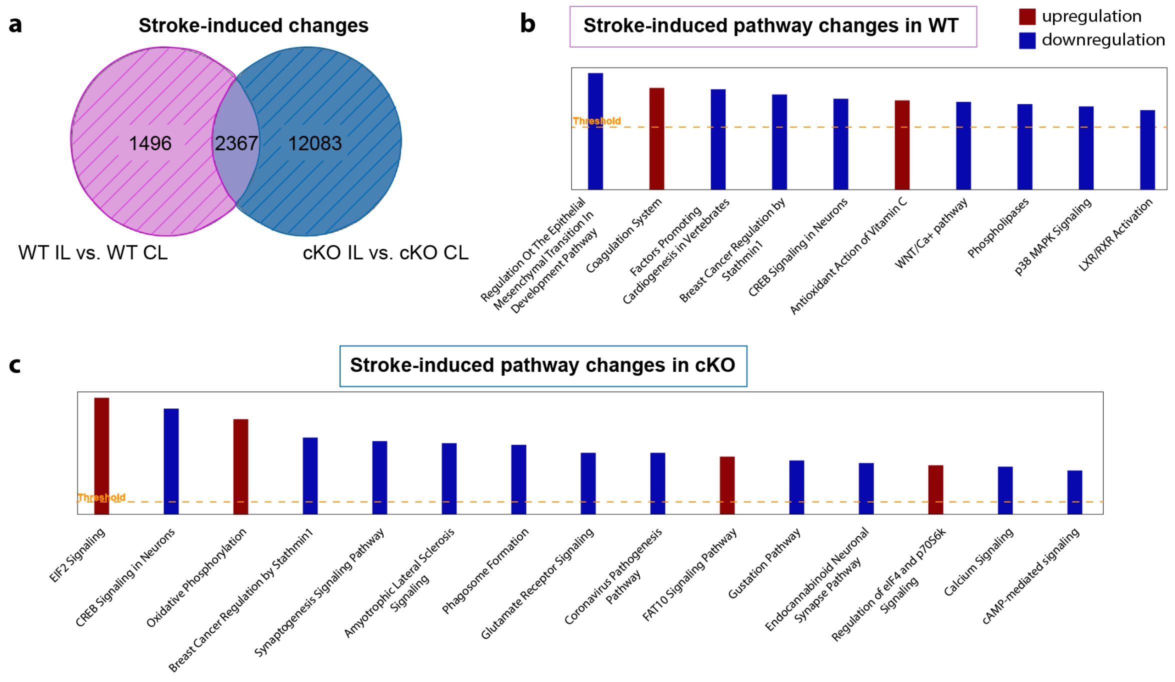

a. Venn diagram showing stroke-induced genes (up- and down-regulated) in WT or cKO microglia (IL vs. CL), with shadowed areas identifying genes of interests for downstream IPA analysis. **b.** Stroke-induced differentially up- and down-regulated pathways in the WT microglia. **c.** Stroke-induced differentially up- and down-regulated pathways in the cKO microglia.

Supplementary Figure 10. Correlation study of cognitive deficits with dendritic spine density.

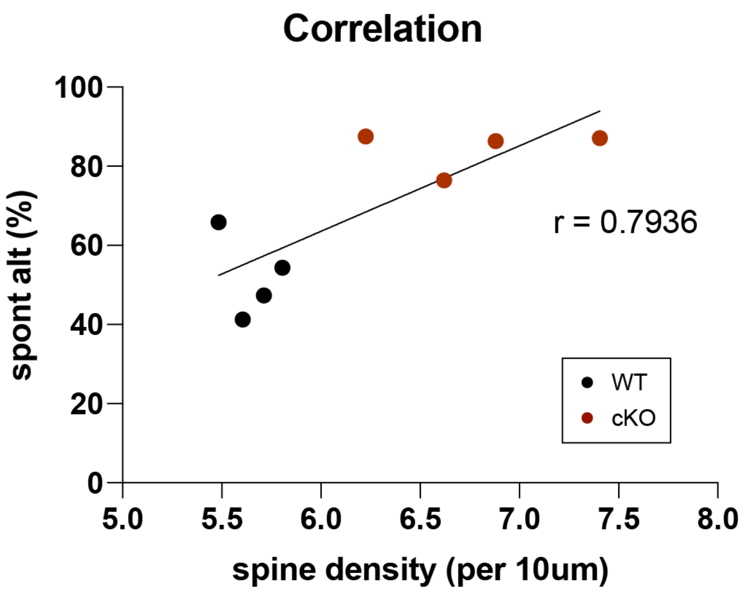

Pearson correlation showed a positive correlation between the spontaneous alternation rates in the Y-maze test, with their dendritic spine density in the Golgi-Cox staining. Matched data were obtained from the same cohort of animals in Figs. 1 and 4. N = 4 for WT and cKO. Pearson coefficient was shown as r.

1
