# Supplementary material for: Elevated microglial oxidative phosphorylation and phagocytosis stimulate post-stroke brain remodeling and cognitive function recovery in mice
Source: Commun Biol. 2022 Jan 11;5:35. doi: 10.1038/s42003-021-02984-4 (PMC8752825; doi:10.1038/s42003-021-02984-4)
Supplement: Supplementary file 3 — Description of Additional Supplementary Files [file 42003_2021_2984_MOESM3_ESM.pdf]

Description of Additional Supplementary Files

File name: Supplementary Data 1
Description: Source data (each Figure is a tab)
