# Supplementary material for: Elevated microglial oxidative phosphorylation and phagocytosis stimulate post-stroke brain remodeling and cognitive function recovery in mice
Source: Commun Biol. 2022 Jan 11;5:35. doi: 10.1038/s42003-021-02984-4 (PMC8752825; doi:10.1038/s42003-021-02984-4)
Supplement: Supplementary file 5 — Reporting Summary [file 42003_2021_2984_MOESM5_ESM.pdf]

nature research

Corresponding author(s): Dandan Sun, Shanshan Song

Last updated by author(s): Oct 24, 2021

Reporting Summary

Nature Research wishes to improve the reproducibility of the work that we publish. This form provides structure for consistency and transparency in reporting. For further information on Nature Research policies, see our Editorial Policies and the Editorial Policy Checklist.

Statistics

For all statistical analyses, confirm that the following items are present in the figure legend, table legend, main text, or Methods section.

n/a	Confirmed	
☐	☒	The exact sample size (*n*) for each experimental group/condition, given as a discrete number and unit of measurement
☐	☒	A statement on whether measurements were taken from distinct samples or whether the same sample was measured repeatedly
☐	☒	The statistical test(s) used AND whether they are one- or two-sided<br>*Only common tests should be described solely by name; describe more complex techniques in the Methods section.*
☒	☐	A description of all covariates tested
☐	☒	A description of any assumptions or corrections, such as tests of normality and adjustment for multiple comparisons
☐	☒	A full description of the statistical parameters including central tendency (e.g. means) or other basic estimates (e.g. regression coefficient) AND variation (e.g. standard deviation) or associated estimates of uncertainty (e.g. confidence intervals)
☐	☒	For null hypothesis testing, the test statistic (e.g. *F*, *t*, *r*) with confidence intervals, effect sizes, degrees of freedom and *P* value noted<br>*Give P values as exact values whenever suitable.*
☒	☐	For Bayesian analysis, information on the choice of priors and Markov chain Monte Carlo settings
☐	☒	For hierarchical and complex designs, identification of the appropriate level for tests and full reporting of outcomes
☐	☒	Estimates of effect sizes (e.g. Cohen's *d*, Pearson's *r*), indicating how they were calculated

Our web collection on statistics for biologists contains articles on many of the points above.

Software and code

Policy information about availability of computer code

Data collection	RNAseq data were collected on the Illumina HiSeq X platform (Illumina, USA). qPCR data were collected on a CFX96 Real-Time PCR Detection System (Bio-rad, USA). Flow cytometry data were collected on a LSR Fortessa flow cytometer (BD Biosciences, USA) running FACS Diva software (BD Bioseceiences, USA). Seahorse data were collected on an XF96 extracellular flux analyzer (Agilent, USA). LC-MS data were collected via a Thermo Vanquish UHPLC and Thermo IDX tribrid mass spectrometer (Thermo Fisher Scientific, USA). Staining images were obtained with Olympus IX83 epifluorescent microscope or Olympus IX81 confocal microscope (Olympus, Japan). TEM images were obtained with a JEOL JEM 1400plus transmission electron microscope (Peabody, MA). Behavioral data were collected with the Fusion behavioral tracking software (Omnitech Electronics, USA) or the Ethovision XT software (Noldus, USA).
Data analysis	RNAseq data were analyzed with Partek Flow 8.0 software (Partek, USA). qPCR data were analyzed with Excel (Microsoft, USA) and plotted in Prism 9.0 software (Graphpad, USA). Flow cytometry data were analyzed with FlowJo software (BD Bioseceiences, USA). Seahorse data were analyzed with Wave v.2.2.0 software (Agilent, USA). LC-MS data were analyzed with Xcalibur Quan Browser 2.7 software (Thermo Fisher Scientific, USA). Fluorescent images and TEM images were analyzed with ImageJ bundled with Java 1.8.0 (NIH, USA). Golgi-Cox stained images were analyzed with Imaris (Bitplane, Switzerland). Behavioral data were analyzed with the Fusion software (Omnitech Electronics, USA) and Excel (Microsoft, USA) before plotted in Prism 9.0 software (Graphpad, USA).

For manuscripts utilizing custom algorithms or software that are central to the research but not yet described in published literature, software must be made available to editors and reviewers. We strongly encourage code deposition in a community repository (e.g. GitHub). See the Nature Research guidelines for submitting code & software for further information.

Data

Policy information about availability of data

All manuscripts must include a data availability statement. This statement should provide the following information, where applicable:

- Accession codes, unique identifiers, or web links for publicly available datasets
- A list of figures that have associated raw data
- A description of any restrictions on data availability

All the data associated with this study are present in the paper or the Supplementary Materials. The RNA sequencing data have been deposited to the Gene Expression Omnibus (GEO) database with experiment series accession number GSE175504.

Field-specific reporting

Please select the one below that is the best fit for your research. If you are not sure, read the appropriate sections before making your selection.

☒ Life sciences ☐ Behavioural & social sciences ☐ Ecological, evolutionary & environmental sciences

For a reference copy of the document with all sections, see nature.com/documents/nr-reporting-summary-flat.pdf

Life sciences study design

All studies must disclose on these points even when the disclosure is negative.

Sample size	Sample size for experiments was determined at the outset and based on power calculations with 80% power and α (two-sided)=0.05, and derived from previous analysis from our laboratory.
Data exclusions	All data were included in the study.
Replication	Experiments were conducted in different sets on different days by different blinded investigators to verify the reproducibility of data collected.
Randomization	Due to the nature of different genotypes, mice could not be randomized into different groups. However, age-matched and sex-matched animals were used whenever possible, and housed in the same facility, and cohorts of mice were bred at the same time. Each group contained roughly equal numbers of male and female animals. All experiments and analysis were conducted blindly whenever possible.
Blinding	Data collection and analysis were conducted blindly whenever possible.

Reporting for specific materials, systems and methods

We require information from authors about some types of materials, experimental systems and methods used in many studies. Here, indicate whether each material, system or method listed is relevant to your study. If you are not sure if a list item applies to your research, read the appropriate section before selecting a response.

Materials & experimental systems

n/a	Involved in the study	
☐	☒	Antibodies
☒	☐	Eukaryotic cell lines
☒	☐	Palaeontology and archaeology
☐	☒	Animals and other organisms
☒	☐	Human research participants
☒	☐	Clinical data
☒	☐	Dual use research of concern

Methods

n/a	Involved in the study	
☒	☐	ChIP-seq
☐	☒	Flow cytometry
☒	☐	MRI-based neuroimaging

Antibodies

Antibodies used	Rabbit polyclonal anti-MBP (Abcam, ab40390), mouse monoclonal anti-Olig2 (Millipore, MABN50), rabbit polyclonal anti-NG2 (Millipore, AB5320), rabbit polyclonal anti-Ki67 (Millipore, ab15580), rabbit polyclonal anti-caspase3 (Cell Signaling Technology, 9662S), rabbit polyclonal anti-H3K9me3 (Abcam, ab8898), mouse monoclonal anti-PSD95 (Cell Signaling Technology, 36233), rabbit polyclonal anti-C1q (Cell Signaling Technology, 6502S), mouse monoclonal anti-SMI32 (BioLegend, 801701), rabbit polyclonal anti-APP (Cell Signaling Technology, 19389S) antibodies were used for immunofluorescent staining primary antibodies, and goat anti-mouse Alexa 546-conjugated IgG (Invitrogen, A-11030), goat anti-rabbit Alexa 488-conjugated IgG (Invitrogen, A-11008), goat anti-mouse Alexa 488-conjugated IgG (Invitrogen, A-11001), and goat anti-rabbit Alexa 546-conjugated IgG (Invitrogen, A-11035) were used as secondary antibodies. BV421-conjugated CD11b (BioLegend, 101235), PerCP-Cy5.5-conjugated CD45 (BioLegend, 103131),

	and APC-conjugated P2RY12 (BioLegend, 848006) antibodies were used for flow cytometry. Biotin-conjugated anti-mouse P2RY12 antibody followed by streptavidin nanobeads (BioLegend, 480114) were used for magnetic separation.
Validation	Rabbit polyclonal anti-MBP (Abcam, ab40390): validated for WB, ICC<br>mouse monoclonal anti-Olig2 (Millipore, MABN50): validated for use in WB, IC, IH & IP.<br>rabbit polyclonal anti-NG2 (Millipore, AB5320): validated for use in IHC and WB<br>rabbit polyclonal anti-Ki67 (Millipore, ab15580): validated for IHC-P, ICC<br>rabbit polyclonal anti-caspase3 (Cell Signaling Technology, 9662S): validated for WB, IP and IHC<br>rabbit polyclonal anti-H3K9me3 (Abcam, ab8898): validated for WB, IHC-P, ICC, ChIP<br>mouse monoclonal anti-PSD95 (Cell Signaling Technology, 36233): validated for WB, IF (Frozen), and IF (ICC)<br>rabbit polyclonal anti-C1q (Cell Signaling Technology, 6502S): validated for WB, IHC (Paraffin), IF (ICC), and Flow Cytometry<br>mouse monoclonal anti-SMI32 (BioLegend, 801701): validated for IHC and WB<br>rabbit polyclonal anti-APP (Cell Signaling Technology, 19389S): validated for IHC<br>BV421-conjugated CD11b (BioLegend, 101235): validated for flow cytometry, ICC, and IHC-F<br>PerCP-Cy5.5-conjugated CD45 (BioLegend, 103131): validated for flow cytometry<br>APC-conjugated P2RY12 (BioLegend, 848006): validated for flow cytometry<br>Biotin-conjugated anti-mouse P2RY12 antibody followed by streptavidin nanobeads (BioLegend, 480114): validated for cell separation

Animals and other organisms

Policy information about studies involving animals; ARRIVE guidelines recommended for reporting animal research

Laboratory animals	Cx3cr1-CreER+/- (wild-type, WT) control mice and Cx3cr1-CreER+/-;Nhe1f/f (Nhe1 cKO) mice of both males or females at 2-3 months of age were used in this study.
Wild animals	N/A
Field-collected samples	N/A
Ethics oversight	All animal studies were approved by the University of Pittsburgh Medical Center Institutional Animal Care and Use Committee, which adhere to the National Institutes of Health Guide for the Care and Use of Laboratory Animals, and reported in accordance with the Animal Research: Reporting In Vivo Experiments (ARRIVE) guidelines. Animals were provided with food and water ad libitum and maintained in a temperature-controlled environment in a 12/12 h light-dark cycle. All efforts were made to minimize animal suffering and the number of animals used.

Note that full information on the approval of the study protocol must also be provided in the manuscript.

Flow Cytometry

Plots

Confirm that:

☒ The axis labels state the marker and fluorochrome used (e.g. CD4-FITC).

☒ The axis scales are clearly visible. Include numbers along axes only for bottom left plot of group (a 'group' is an analysis of identical markers).

☒ All plots are contour plots with outliers or pseudocolor plots.

☒ A numerical value for number of cells or percentage (with statistics) is provided.

Methodology

Sample preparation	Mice were euthanized with overdose of CO2 and transcardially perfused with ice-cold saline. CL or IL hemispheres were collected separately, and single cell suspensions were prepared using a neural tissue dissociation kit with the gentleMAC Octo Dissociator (Miltenyi Biotech Inc., Germany). Myelin was removed using the 30/70 Percoll gradient method before further processing.
Instrument	LSR Fortessa flow cytometer (BD Biosciences, USA) running FACS Diva software (BD Biosceiences, USA)
Software	FlowJo software (BD Biosceiences, USA) were used for data analysis.
Cell population abundance	Gated under the CD11b+/CD45low-med population, abundance of microglial cells were further verified by P2RY12+ population. Approximately ~85% of the CD11b+/CD45low-med population were P2RY12+, while microglial cell counts identified by CD11b+/CD45low-med population or the CD11b+/CD45low-med/P2RY12+ population displayed a strong correlation of r = 0.9638 (p < 0.0001), as verified in our previous study (Song et al, Glia, 2018).
Gating strategy	Gating of each channel was determined by comparing to non-stained cell control and single-color stained compensation beads.

☒ Tick this box to confirm that a figure exemplifying the gating strategy is provided in the Supplementary Information.
